# Three Dimensional Culture of Human Renal Cell Carcinoma Organoids

**DOI:** 10.1371/journal.pone.0136758

**Published:** 2015-08-28

**Authors:** Cynthia A. Batchelder, Michele L. Martinez, Nadire Duru, Frederick J. Meyers, Alice F. Tarantal

**Affiliations:** 1 California National Primate Research Center, University of California Davis, Davis, CA, United States of America; 2 Department of Internal Medicine, School of Medicine, University of California Davis, Davis, CA, United States of America; 3 Comprehensive Cancer Center, University of California Davis, Davis, CA, United States of America; 4 Department of Pediatrics, School of Medicine, University of California Davis, Davis, CA, United States of America; 5 Department of Cell Biology and Human Anatomy, School of Medicine, University of California Davis, Davis, CA, United States of America; Michigan Technological University, UNITED STATES

## Abstract

Renal cell carcinomas arise from the nephron but are heterogeneous in disease biology, clinical behavior, prognosis, and response to systemic therapy. Development of patient-specific *in vitro* models that efficiently and faithfully reproduce the *in vivo* phenotype may provide a means to develop personalized therapies for this diverse carcinoma. Studies to maintain and model tumor phenotypes *in vitro* were conducted with emerging three-dimensional culture techniques and natural scaffolding materials. Human renal cell carcinomas were individually characterized by histology, immunohistochemistry, and quantitative PCR to establish the characteristics of each tumor. Isolated cells were cultured on renal extracellular matrix and compared to a novel polysaccharide scaffold to assess cell-scaffold interactions, development of organoids, and maintenance of gene expression signatures over time in culture. Renal cell carcinomas cultured on renal extracellular matrix repopulated tubules or vessel lumens in renal pyramids and medullary rays, but cells were not observed in glomeruli or outer cortical regions of the scaffold. In the polysaccharide scaffold, renal cell carcinomas formed aggregates that were loosely attached to the scaffold or free-floating within the matrix. Molecular analysis of cell-scaffold constructs including immunohistochemistry and quantitative PCR demonstrated that individual tumor phenotypes could be sustained for up to 21 days in culture on both scaffolds, and in comparison to outcomes in two-dimensional monolayer cultures. The use of three-dimensional scaffolds to engineer a personalized *in vitro* renal cell carcinoma model provides opportunities to advance understanding of this disease.

## Introduction

Kidney cancer is one of the ten most common malignancies in the United States and is increasing in frequency, due in part to greater prevalence of putative risk factors including smoking, obesity, and hypertension, as well as increased detection resulting from improvements in diagnostic imaging [1]. Within the broad classification of kidney cancers, renal cell carcinoma (RCC) accounts for approximately 85% of all cases and greater than 90% of all renal malignancies. The annual financial burden for treating RCC is over $4 billion in the United States alone and continues to rise with over 60,000 new cases diagnosed annually [2]. This diverse group of cancers includes clear cell, papillary, chromophobe, collecting duct, and medullary subtypes and is associated with challenges in defining prognosis and in predicting response to therapy. The RCC subtypes share the nephron as a common site of origin but differ in disease biology, clinical behavior, prognosis, and response to therapy [3]. At present, the RCC subtypes can be distinguished histologically but identification of specific biomarkers for screening, diagnosis, and to predict therapeutic response would significantly improve treatment approaches and outcomes. Development of patient-specific *in vitro* organoid models for RCC that efficiently, faithfully, and economically reproduce the *in vivo* phenotype are essential for the development of targeted, personalized therapies for this diverse group of cancers.

*In vitro* studies of RCC are challenging due to the complex three-dimensional (3D) architecture of the kidney. The current standard for RCC culture involves primary [4–6] or immortalized cells grown on conventional two-dimensional (2D) tissue culture plastic. In many instances, the phenotype of the parental tumor from which a 2D cell line has been established is unknown, or the culture fails to maintain the primary phenotype over time [7]. Issues of validity in 2D *in vitro* studies are not unique to RCC, but also pose challenges in studies to predict the success or failure of new drug candidates and to predict nephrotoxicity [8, 9]. Emerging 3D culture methods will likely improve the ability to model tumor behavior in culture as this technique provides a supportive milieu although scaffolds that can support growth and the nascent phenotype are needed [10–14].

Our studies have previously demonstrated that decellularized kidneys of all age groups provide a natural extracellular matrix (ECM) with sufficient structural properties to support migration of cells from kidney explants to repopulate the scaffold in an age-dependent manner [15], and the ability to provide spatial and organizational influences on human embryonic stem cell migration and differentiation [16–18]. The goals of the current study were to: (1) develop improved 3D scaffold and culture methods for the *in vitro* study of RCC, and (2) assess scaffold support of RCC organoids with maintenance of the parental tumor phenotype. These studies demonstrate that individual tumor phenotypes could be maintained under the 3D culture conditions as described, and that the scaffolds provide a means to support the growth and development of organoids with the same phenotypic features of the parental tumor.

## Materials and Methods

### Specimens

No human subjects were involved in the study. The UC Davis Comprehensive Cancer Center, which is funded by the National Cancer Institute (NCI), has a biorepository that provides anonymized specimens to investigators through university approved practices and protocols (http://www.ucdmc.ucdavis.edu/cancer/research/sharedresources/specimen.html). No animal subjects were involved in the study. A biorepository of previously obtained decellularized rhesus monkey kidney sections were used for these studies; kidneys were obtained through the tissue procurement program (www.cnprc.ucdavis.edu/our-services).

The UC Davis Comprehensive Cancer Center's Biorepository Shared Resource provides high quality, well-characterized cancer-related human tissue specimens and biological materials to researchers. Anonymized resected tumor sections (N = 25) and corresponding non-tumor (distal to the tumor) (N = 22) specimens were obtained. Specimens collected were used for primary cell cultures, snap frozen in liquid nitrogen for molecular analysis, and sections collected in 10% buffered formalin.

### Primary Cultures

Specimens were finely minced under sterile conditions in endothelial growth medium (EGM2; Lonza, Walkersville, MD), which we have previously shown in preliminary studies to support the growth of multiple renal cell phenotypes in culture (data not shown). Minced tissue was then dissociated by incubation with collagenase Type IV (5 mg/ml, Life Technologies, Grand Island, NY) at 37°C for 40 to 50 minutes with vortexing at 10 minute intervals. Specimens were maintained at a size that could be transferred with a serological pipette, then additional medium was added (1:1 ratio) to halt the dissociation process. Following centrifugation, the supernatant containing collagenase was discarded and the cell pellet re-suspended in EGM2 for culture on standard tissue-culture dishes (Sigma-Aldrich, St. Louis, MO). Medium was changed at 3-day intervals once cells were adherent (48–72 hours) and cultures maintained at 37°C, 5% CO_2_ until 80% confluent. Cultures were then utilized for 3D experiments (passage 0–2).

### RT-PCR Tumor Gene Panel

Fresh tissues snap-frozen in liquid nitrogen were used to isolate RNA with the AllPrep DNA/RNA mini kit (Qiagen, Valencia, CA) and cDNA prepared following DNase I treatment (Qiagen) with random primers (Ambion, Life Technologies) and the Sensiscript Reverse Transcriptase kit (Qiagen). NextBio (www.nextbio.com), a repository of data from genomic studies and patient molecular profiles, was used to assess potential markers for RT-PCR analysis. The markers selected from NextBio included: Carbonic anhydrase 9 (CA9), Egl nine homolog 3 (EGLN3), Ectonucleotide pyrophosphatase/phosphodiesterase family member 3 (ENPP3), Fatty acid binding protein 7 (FABP7), KISS1-derived peptide receptor (KISS1R), Lysyl oxidase (LOX), and Neuronal pentraxin 2 (NPTX2). This panel of markers was expanded to include the renal stem and progenitor cell markers Octamer-binding transcription factor 4 (OCT4), Oxidative-stress responsive 1 (OSR1), Sine oculis homeobox homolog 1 (SIX1), and Sine oculis homeobox homolog 1 (SIX2). RT-PCR was performed to assess the expression of these markers in tumor and non-tumor samples. The housekeeping gene, Glyceraldehyde-3-phosphate dehydrogenase (GAPDH) was used as the internal control for CA9, EGLN3, ENPP3, FABP7, LOX, and NPTX2, which were assessed with Taqman probe-based RT-PCR (Life Technologies). Elongation factor-1 alpha (EF1α) was used as the internal control for OCT4, OSR1, SIX1, and SIX2, which were assessed with SYBR Green dye-based RT-PCR (Qiagen) and primer sets published previously [13]. Relative gene expression was compared with the ΔΔC_T_ method utilizing normal human kidney cDNA prepared from human kidney total RNA (Life Technologies).

### 3D Organoid Culture

Two 3D scaffolds were used: stored rhesus monkey decellularized sections of renal scaffolds (renal ECM) [15] and a polysaccharide scaffold (PSS) (GroCell-3D, Molecular Matrix Inc., Davis, CA). As previously shown, the renal ECM retained the vascular, tubular, and glomerular compartments of the native kidney with removal of greater than 99% of all other non-ECM proteins including collagenase. The PSS contains pores of an approximate 200-μm diameter and 500–700 μm in length (S1 Fig). Due to size and quality limitations of the resected specimens received, cells were plated in 2D to allow expansion prior to plating on 3D scaffolds. Low passage (0–2) cells from individual tumor or non-tumor cultures (from each individual specimen) were seeded on scaffolds in order to create a unique and personalized cell-scaffold construct. Standard 2D cultures on tissue culture plastic were utilized as cell culture controls. Cells from each resected tumor or cells distal to the tumor were plated (approximately 1x10^6^ cells/8 mm scaffold diameter) by gently applying cells to the scaffold surface in 20-μl aliquots of medium. Cell-scaffold constructs composed of tumor or non-tumor from a given specimen were cultured in 24-well plates for 5±1 hours to allow cell adherence prior to addition of EGM2. Medium was changed at 3-day intervals throughout the culture period.

### Analysis of Cell-Scaffold Constructs

Cell-scaffold constructs were collected at 1, 2, or 3-week intervals and fixed in 10% buffered formalin for 4 hours, transferred to 70% ethanol, then processed for paraffin embedding. Paraffin blocks were exhaustively sectioned at 5-μm thickness for histological analysis. At 10-section intervals, additional formalin-fixed, paraffin-embedded (FFPE) sections were collected for molecular analysis by changing sectioning thickness to 10 μm and collecting two sections in microcentrifuge tubes for PCR. Tubes for molecular analysis were stored at ≤-80°C until processing.

### Construct Histological Analysis

For each cell-scaffold construct, every 10^th^ section was stained with hematoxylin and eosin (H&E) to assess cell infiltration and morphology. Sections were viewed with an Olympus BX61 microscope (Olympus, Center Valley, PA) and images captured with MetaMorph Image Analysis Software (Molecular Devices, Sunnyvale, CA). Morphology of cells within scaffolds was characterized as: (1) non-adherent or loosely attached organoids, (2) adherent, epithelial-like cells lining edges and lumens, or (3) single cells. Immunohistochemistry was performed on selected adjacent sections for Cytokeratin (wide-spectrum, rabbit polyclonal, Abcam) and Vimentin (mouse monoclonal V9, Sigma-Aldrich) following previously established protocols [13]. Staining for the RCC antigen was also conducted according to established protocols [14].

### Construct Molecular Analysis

DNA and RNA were isolated from FFPE sections using the AllPrep DNA/RNA FFPE kit (Qiagen). Isolated RNA was treated with DNase I (Qiagen) to eliminate potential DNA contamination prior to downstream analysis. cDNA was synthesized using random primers (Ambion, Life Technologies) and the Sensiscript Reverse Transcriptase kit (Qiagen). For each tumor, signature genes found to be upregulated from normative range kidney standards were identified by the tumor gene panel screen and utilized to monitor cell phenotype over time in 2D and 3D cultures. PCR for selected markers was carried out with appropriate SYBR or Taqman assay protocols as described above. Relative changes in gene expression were analyzed by the comparative ΔΔC_T_ method normalized to the cells on the day of seeding.

### Statistical Analysis

Results of qPCR gene expression analysis were expressed as the mean ± standard error of the mean (SEM). A multivariate analysis of variance (MANOVA) was conducted on log-transformed gene expression data to assess the following relationships: tumor samples versus normal human kidney standards; tumor samples versus non-tumor samples from the same kidney; and to test for differences of tumor subtype. When the MANOVA was significant, posthoc t-tests were used to assess significance of changes in gene expression for individual genes. The chi-squared test was used to assess significant differences in the frequency of epithelial or organoid outcomes in cell-scaffold constructs.

## Results

### Patient Demographics

The gender, age, and racial demographics of individuals from which anonymized specimens were received (N = 25 tumor, N = 22 non-tumor site) are summarized in Table 1. Patients ranged in age from 31 to 85 years (mean 61 years, median 59 years).

**Table 1:** Demographics.

**Sex**	**N**	**Race / Ethnicity**	**N**
Male	18 (72%)	White, non-Hispanic	18 (72%)
Female	7 (32%)	Black / African-American	1 (4%)
** Age (Years)**	**N**	American Indian / Alaska Native	1 (4%)
≤45	2 (8%)	Asian	2 (8%)
46–55	8 (32%)	Not reported	3 (12%)
56–65	5 (20%)		
66–75	6 (24%)		
≥76	4 (16%)		

### RCC Subtype and Histopathology Distribution

Clinical pathology reports for each tumor were provided by the UC Davis Comprehensive Cancer Center and are summarized in Table 2. Specimens included the three most common RCC subtypes: clear cell (60%), papillary (24%), and chromophobe (4%); and correlated with reported population frequencies of these subtypes [19–21]. Nearly 50% of the tumors were classified as pT1a with 16% as pT2a. Mean tumor size at resection was 5.1 cm with a range of 0.9 to 12.0 cm. More than half of the tumors (60%) were less than 5.0 cm in size. The predominant Fuhrman nuclear grade was 2 (40%) followed by 3 (15%) and 1 (12%). H&E staining indicated the specimens were representative of the expected morphology for each RCC subtype. Non-tumor specimens from the same kidneys were frequently observed with atypical histopathology including hypercellularity and the presence of epithelial nests. Immunohistochemistry was completed on FFPE sections to further characterize the phenotype of each specimen. Cytokeratin and vimentin co-expression was observed in 70% of tissues and was not correlated with a specific RCC subtype. Remaining specimens were vimentin-positive and cytokeratin-negative. In non-tumor specimens, vimentin staining was noted in mesangial cells of the glomeruli with cytokeratin staining found in some, but not all, tubules. Double-positive cells were typically not observed in non-tumor tissues with the exception of the parietal epithelium of Bowman’s capsule and rare tubule segments. A subset of specimens evaluated (9/10) expressed RCC antigen with varied intensity that was not correlated with tumor subtype. Proximal tubules of non-tumor tissues also routinely stained positive for RCC antigen (data not shown).

**Table 2:** Renal Cell Carcinoma (RCC) Subtype and Classification.

**RCC Subtype**	**N**	**Tumor Grade**	**N**
Clear Cell	15 (60%)	1	3 (12%)
Papillary	6 (24%)	2	10 (40%)
Chromophobe	1 (4%)	3	4 (16%)
Oncocytoma	2 (8%)	4	2 (8%)
Mixed	1 (4%)	ND	6 (24%)
**Classification**	**N**	**Size (cm)**	**N**
pT1a	12 (48%)	< 5	15 (60%)
pT1b	2 (8%)	≥ 5 to < 7	2 (8%)
pT2a	4 (16%)	≥ 7 to < 10	4 (16%)
pT2b	1 (4%)	≥ 10	3 (12%)
pT3a	2 (8%)	ND	1 (4%)
ND	4 (16%)		

Mixed^:^ Separate foci of clear cell and papillary RCC noted; Size: Range 0.9 to 12.0 cm, Mean 5.1 cm, Median 3.9 cm

ND = not determined.

### RCC Gene Expression

**Fig 1:**
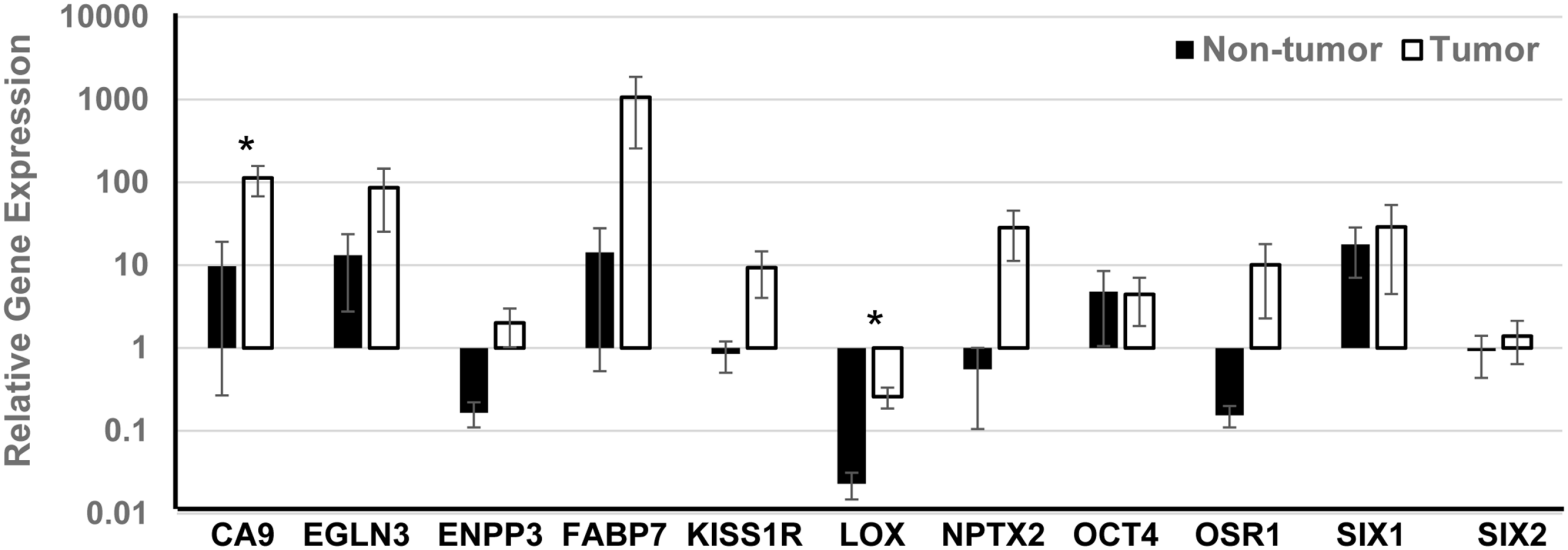
Relative gene expression in RCC tumor and non-tumor samples. Gene expression relative to normal human kidney cDNA was calculated from RCC tumor (N = 22) and non-tumor (N = 18) samples and presented as the mean ± standard error of the mean (SEM); *p<0.05. Relative to grossly normal kidney, all genes were upregulated more than 2-fold except for LOX (downregulated) and SIX2 (upregulated 1.4 fold) in tumor samples. Expression was also upregulated in non-tumor samples for many genes. Differences between tumor and non-tumor samples were not significant with the exception of CA9 and LOX (*p*<0.05).

Tumor and non-tumor specimens from each case were screened with a panel of markers previously identified to be upregulated in RCC. An initial search for a gene upregulated (≥3-fold increase in gene expression compared with non-tumor kidney specimens) across all cases was explored with the goal of identifying a single marker that could be used to assess the presence of RCC over time in culture. Relative gene expression data from individual samples was highly variable with no single gene consistently upregulated across cases or within an RCC subtype. Genes upregulated with the greatest frequency included CA9 (59.1%), NPTX2 (54.5%), and KISS1R (40.9%). When averaged across specimens, expression increased in RCC samples for all genes in the panel except LOX, which was downregulated. The genes upregulated greater than 5-fold compared with control kidney standards included CA9, EGLN3, FABP7, KISS1R, NPTX2, OCT4, and SIX1. These genes, with the exception of KISS1R and NPTX2, were also upregulated in non-tumor samples from RCC-affected kidneys (Fig 1). Differences in gene expression across all cases when comparing tumor and non-tumor samples were not statistically significant with the exception of CA9 and LOX (*p*<0.05). Given the RCC heterogeneity demonstrated in these results, two to four markers were selected specific to each case as a unique and personalized “signature” for *in vitro* analysis of 3D constructs engineered with cells from a given tumor.

### RCC *In vitro* Cultures

**Fig 2:**
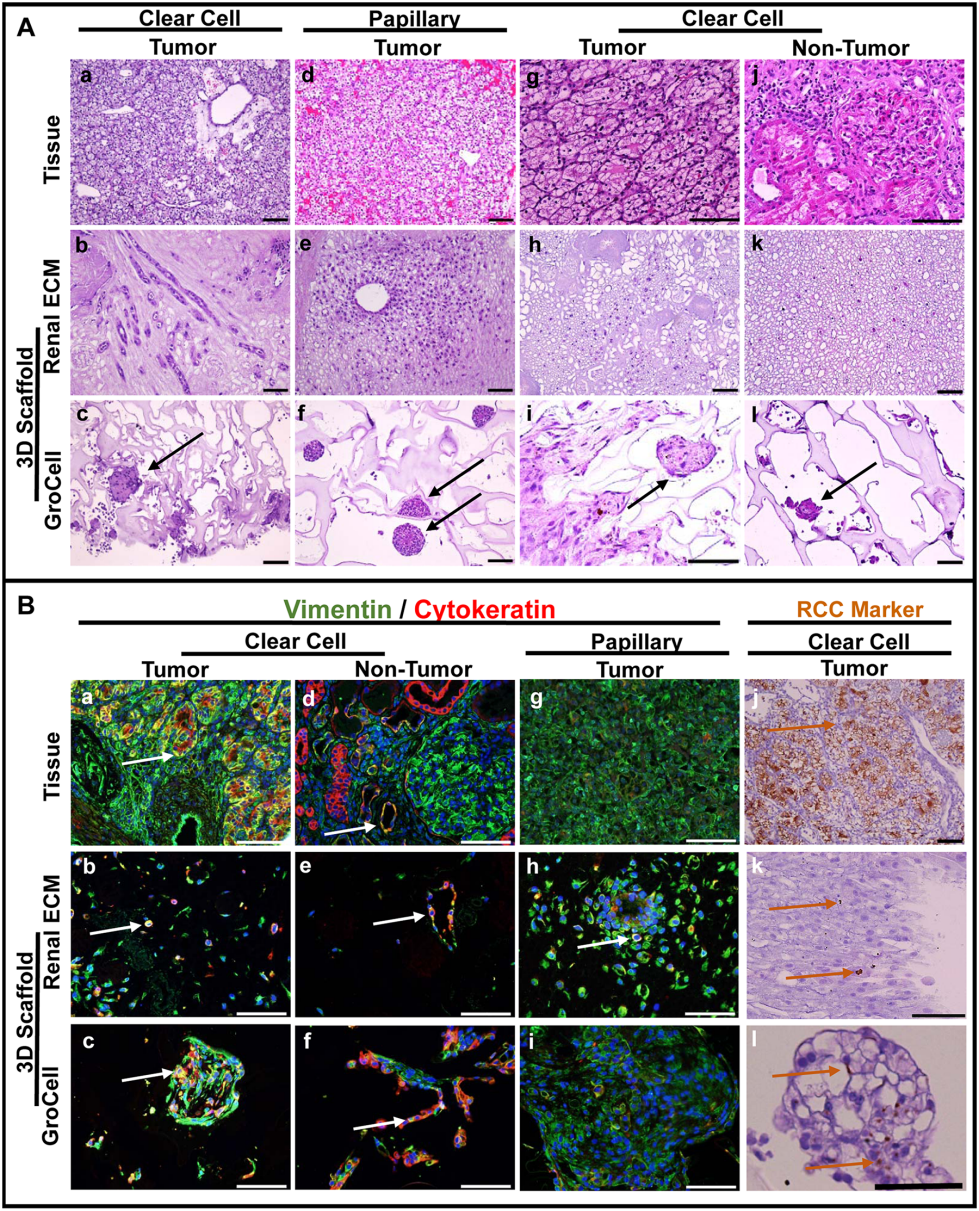
Phenotypic comparison of 3D RCC-scaffold constructs with parental tumor characteristics. **A.** Hematoxylin and eosin (H&E) staining of parental tissue and corresponding RCC 3D constructs with renal ECM or polysaccharide scaffolds (PSS). Representative examples shown include clear cell (**a-c**) and papillary (**d-f**) RCC subtypes. Tumor (**g-i**) and non-tumor (**j-l**) tissue and 3D constructs from a single clear cell RCC-affected kidney are also shown. Regardless of tumor subtype, RCC typically repopulated medullary regions of the renal ECM, specifically the pyramids and medullary rays. RCC in the PSS were predominantly found as heterogeneous organoids (black arrows) that were free-floating within, or loosely attached to, the scaffold. **B.** Immunohistochemical staining for vimentin (green), cytokeratin (red), and the RCC Marker (brown). Representative examples are shown from clear cell tumor (**a-c**) and non-tumor (**d-f**) tissue from the same kidney; and papillary (**g-i**), and clear cell (**j-l**) RCC. Co-expression of cytokeratin and vimentin (white arrows) was noted in 70% of parental tumor tissues with remaining tissues vimentin-positive. Tumor-derived cell-scaffold constructs typically expressed the cytokeratin / vimentin staining pattern of the parental tissue, although increased vimentin expression was noted on renal ECM. Co-expression of these markers was only observed in parietal epithelial cells of Bowman’s capsule in histologically normal non-tumor tissues. In rare instances, non-tumor tissues contained tubules with double-positive cells (**d**). Strong RCC Marker (brown arrows) expression was observed in 90% of specimens, in proximal tubules of non-tumor tissue, and was maintained in some, but not all, 3D RCC constructs (**k, l**). Scale bars = 100 μm.

**Fig 3:**
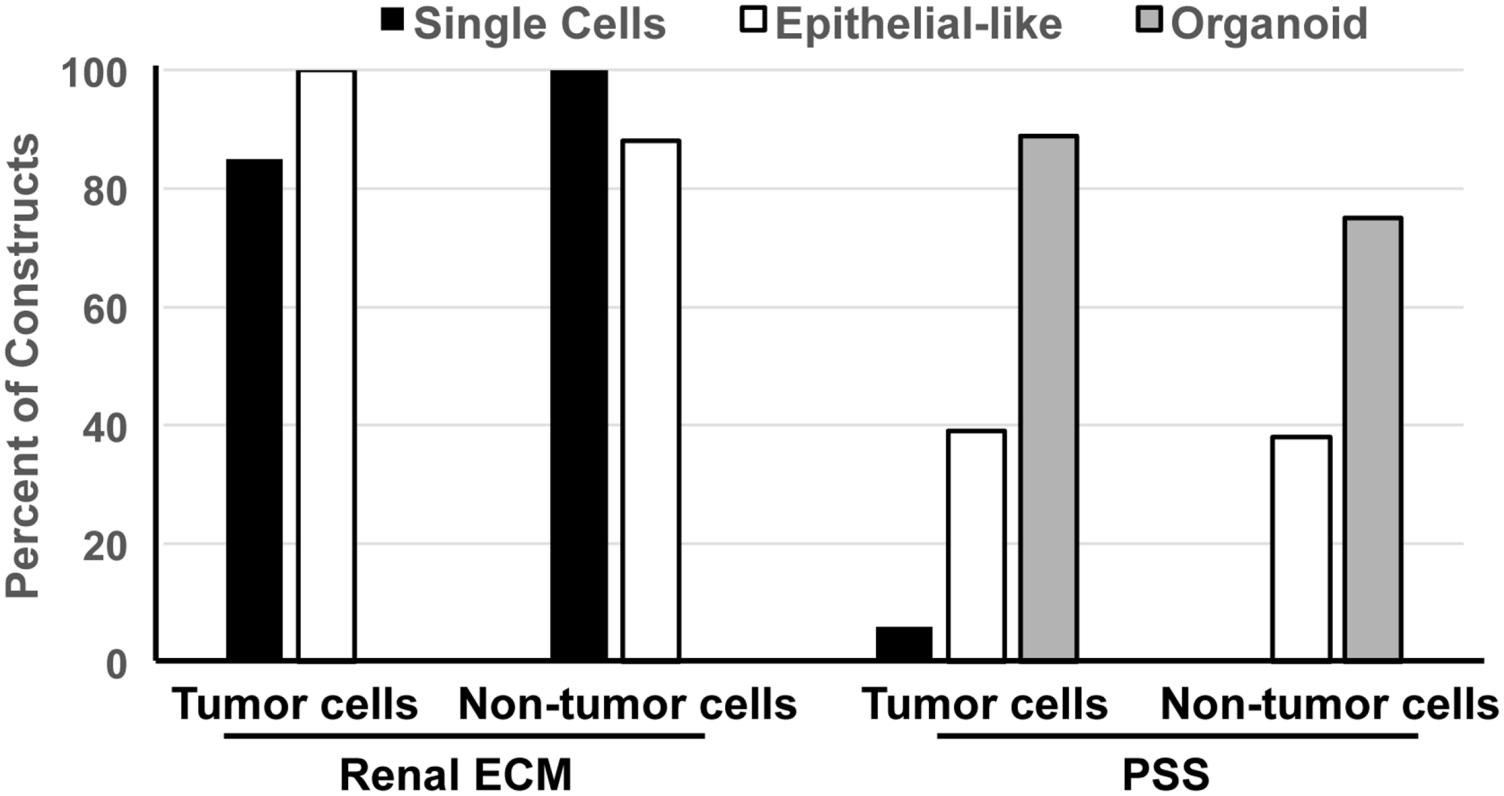
RCC organoid formation in 3D is dependent on scaffold type. Non-parametric characterization of cell morphology in 3D tissue engineered constructs. Some constructs contained cells in more than one classification. Tumor (N = 15) and non-tumor (N = 8) cells were found as single cells or lining some tubules of the renal pyramid and medullary rays in 3D cell-renal ECM constructs. In the 3D PSS constructs, tumor (N = 22) and non-tumor (N = 8) cells were typically observed as loosely-attached organoid clusters, although some cells were found lining scaffold lumens. Scaffold impact on cell morphology was significant (*p*<0.001).

Primary cultures were established from 20/25 tumor specimens and 22/22 non-tumor samples. Those specimens associated with poor derivation of cell culture included a predominance of adipose or necrotic regions in the sample. In general, dissociated cells from non-tumor samples attached to the plate surface and grew more rapidly than cells from RCC in primary 2D cultures. When cultured in 3D scaffolds, RCC proliferation was observed at week 1 with continued growth and expansion evident over the 3-week culture period. Sections of 3D constructs were analyzed by H&E to assess cell frequency, location, morphology, and to compare with initial specimen histology (Fig 2A). Morphology and location of RCC in the renal ECM scaffold was consistent across all specimens with cells observed as individual, epithelial-like cells in the medullary regions or lining tubules or vessel lumens in renal pyramids and medullary ray locations. RCC consistently failed to repopulate glomeruli or cortical tubules of renal ECM scaffolds. In contrast, the morphology of RCC cultured in the PSS included organoid aggregates as a predominant feature, with some cells found lining lumens in an epithelial-like arrangement. Organoid aggregates were observed in the PSS in both tumor (89%) and non-tumor (75%) constructs but typically larger in size when cells from the RCC were used (Fig 3). The RCC organoids were frequently free-floating or loosely attached to the PSS lattice and were not observed to form in renal ECM scaffolds.

### RCC Phenotype in 3D Constructs: Immunohistochemistry

The molecular phenotype of RCC within the scaffolds was assessed by immunohistochemistry for cytokeratin, vimentin, and RCC antigen and compared with expression in the corresponding tumor or non-tumor specimens from which cells were derived (Fig 2B). The cytokeratin / vimentin staining pattern of the parental tumor was typically maintained in 3D constructs although cytokeratin expression was observed more frequently in cells on renal ECM than in the PSS. Notably, cells from non-tumor samples were frequently double positive for cytokeratin and vimentin in 3D cultures with both scaffold types despite the presence of few double positive cells observed in the parental sample. RCC Marker expression was reduced in 3D cultures compared with parental tumor or non-tumor samples, but maintained more frequently in the PSS constructs (80%) than renal ECM constructs (50%).

### RCC Phenotype in 3D Constructs: Gene Expression

**Fig 4:**
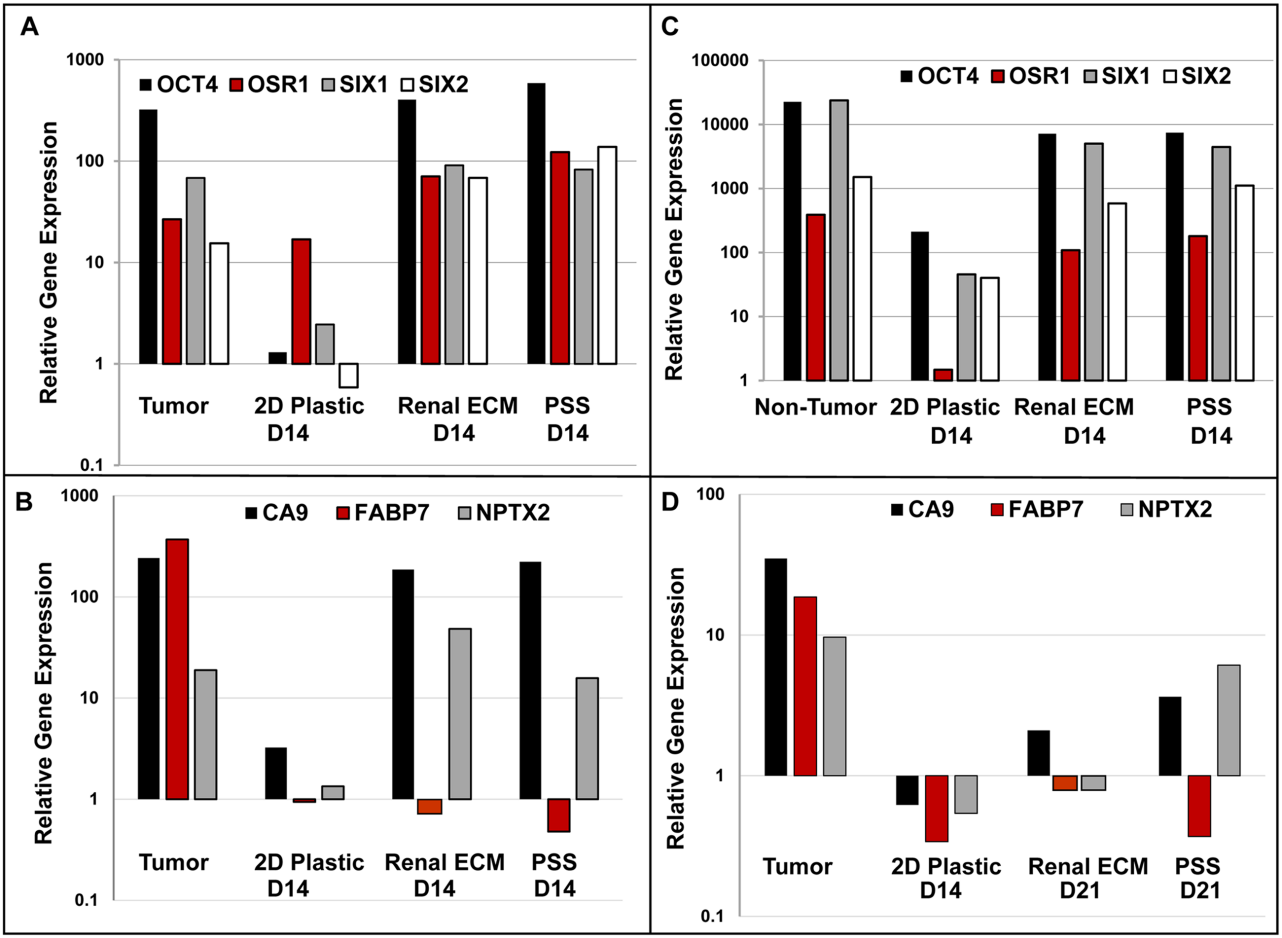
RCC gene expression signature is maintained over time in 3D, but not 2D, cultures. Relative expression of selected tumor signature genes in tissue (tumor, non-tumor) and after 14 or 21 days in monolayer culture (2D plastic) or on 3D scaffolds (Renal ECM or PSS). PCR samples were run in triplicate to ensure accuracy with appropriate PCR reagents and negative transcriptase controls. Representative examples are shown from papillary (**A**) and clear cell (**B, D**) tumors, and non-tumor clear cell specimens (**C**). Because of the variation in gene expression signature from one biological replicate (e.g., patient sample) to the next, pooling of data was not appropriate. Instead, this analysis focused on the degree to which the original tumor or non-tumor gene expression pattern was maintained over time in culture across all patients (N = 22 tumor, N = 8 non-tumor 3D cultures). In all cases, expression of the signature gene set was more frequently maintained in 3D cultures. Differences in gene expression were not detected between renal ECM and the PSS. Despite strong expression in tumor tissues, expression of FABP7 was not maintained with any culture condition tested.

Genes upregulated in individual RCC specimens were selected to monitor the phenotype by RT-PCR in 3D cell-scaffold constructs. Expression of tumor signature genes was consistently maintained in 3D cultures for up to 21 days, with loss of expression of tumor genes noted in 2D culture conditions (Fig 4). For a given RCC, differences in gene expression were not detected between renal ECM and the PSS. FABP7 expression was not maintained *in vitro* under any culture conditions tested suggesting that additional substrates or growth factors may be necessary to maintain expression of this gene. When non-tumor cells were plated on 3D scaffolds, expression patterns were similar to RCC from the same kidney suggesting that a small population of tumor cells with a proliferative advantage thrived in 3D culture from the non-tumor sample.

## Discussion

The barriers to development of more effective treatment regimens for RCC include a lack of distinguishing biomarkers, subtype differences, heterogeneity of underlying acquired or hereditary genetic mutations, and inter-patient diversity [22, 23]. These factors also complicate *in vitro* studies of tumor biology and drug development, and highlight the need for new strategies to address these issues. *In vitro* 3D culture conditions for RCC which faithfully reproduce the molecular and histological phenotype of the parental tumor more precisely than standard 2D culture conditions, and which can be correlated with patient outcomes, may provide a solution for basic studies of tumor biology and to efficiently assess targeted and patient-specific personalized therapies.

Gene expression analysis can be a useful tool to provide insight into tumor biology, prognosis, or to allow sub-grouping of a clinically diverse group of cancers for more effective treatment strategies. Previous RCC gene expression studies have documented the heterogeneity in RNA expression across samples and identified various sets of 20+ genes with potential predictive power to recognize aggressive, metastatic tumors from less aggressive forms of the disease [24–26]. To facilitate *in vitro* studies where maintenance of the cellular phenotype over time in culture is essential, we chose to identify a smaller set of select genes upregulated in individual samples as a personalized signature for a given tumor. With this method, a large gene panel can effectively focus on a few signature genes, which uniquely represent a given tumor and can be utilized to ensure individual cultures maintain the tumor phenotype over time and across studies. Intra-tumor heterogeneity, as described in studies of gene expression in RCC biopsy samples [27] or in terms of cell composition, was not assessed in this study and will be important to understand in the context of *in vitro* culture in future studies. The resected specimens, while typically larger than a standard biopsy sample, were not inclusive of the entire tumor; future studies will be necessary to assess the size of the specimen needed to accurately represent the bulk tumor cell or gene expression profile. Similarly, initial 2D expansion was often necessary to isolate cells from small specimens. Further studies to establish 3D tumor constructs directly from resected specimens may provide additional insights regarding intra-tumor cellular heterogeneity.

Previous *in vitro* RCC studies have relied primarily on 2D monolayer cultures of primary or immortalized cell lines [6]. While these cultures may retain DNA copy number profiles of the parental tumor [28, 29], loss of cellular heterozygosity has been reported and relevance to the *in vivo* setting is not reflected in the correlation of *in vitro* drug development or kidney toxicity screens to patient outcomes [8]. Advances in tissue engineering with a focus on the kidney suggest application of these 3D culture methods to studies on renal development or disease may enhance *in vitro* models by more closely recapitulating *in vivo* interactions with other cells and ECM molecules, therefore offering more opportunities for long-term culture maintenance. Several groups have assessed 3D culture conditions for primary renal cells or RCC with various substrates including small intestine submucosa [30] and Matrigel [31, 32], or as suspension cultures of spheroid aggregates [9, 33, 34]. Tumor grafts of fresh human specimens implanted in immunodeficient mice (“patient derived xenografts”) have been shown to retain the morphology and characteristics of parental tumors [35] and have also been utilized to study specific drug activity against RCC [7, 36]. Identifying new ways to utilize natural scaffolds and matrices that can demonstrate maintenance of the 3D architecture and cellular heterogeneity of tumor biology will be important to accurately and efficiently assess drug responses [37]. Organoids, which contain multiple organ-specific cell types and architectural similarities to the native organ, have been developed from small intestine [10–12] and other organs including the kidney [8, 9] and emphasize the importance of these *in vitro* culture systems to study effective therapies specific to the patient and their disease. The development of 3D organoid protocols for RCC provides an *in vitro* resource for studies on RCC in general and for drug development and kidney toxicity screens in particular.

The ability of 3D scaffolds to support RCC growth and to maintain the phenotype over time was assessed in this study with renal ECM from decellularized kidneys and a patented PSS. These results indicate that the scaffold material significantly influences cell morphology but does not impact gene expression signatures of individual tumors. Gene expression phenotypes of individual RCC were typically lost in 2D cultures but could be maintained for up to 21 days in 3D cultures as noted. Maintenance of a signature gene expression pattern was largely uniform across the heterogeneous set of tumors analyzed, with the exception of FABP7, a gene for which expression was not maintained from any tumor or under any culture condition tested.

Although gene expression phenotypes were maintained with both 3D scaffolds, the organoid nature of RCC growth in the PSS suggests this natural material may be more efficient for long-term studies to assess interactions of stromal or non-tumor cells with malignant cells, and as a tool to predict therapeutic responses. In contrast, the unique interaction of RCC with the medullary components of the renal ECM suggests this interface may be valuable for future studies of the metastatic origins of RCC, and specific tubular characteristics that may influence tumor proliferation. Further development of 3D culture strategies will permit high throughput opportunities to study patient-specific interactions of malignant cells with other cell types within the niche including stromal, epithelial, and endothelial cells [7, 14, 32]. Patients with advanced RCC that do not respond to standard chemotherapy may require more personalized treatment, which targets different tumor subtypes as well as primary RCC. Three-dimensional RCC *in vitro* models may provide a unique approach for developing and screening chemotherapeutic agents that target all tumor subtypes. These *in vitro* models may also reduce the number of *in vivo* studies required for drug development and screening.

The studies described herein demonstrate that RCC cell-scaffold 3D constructs are useful for engineering personalized RCC *in vitro* models, and provide important opportunities for the study of pathogenesis, progression, and drug screening that may improve outcomes for RCC patients.

## Supporting Information

S1 Fig Scaffold morphology. **A.** Renal ECM from decellularized rhesus monkey kidney retains tubular and vascular lumens as well as glomerular compartments. Scale bar = 100 μm. **B.** SEM of polysaccharide scaffold (PSS) with pores connected of 500–700 μm. Scale bar = 500 μm.
(TIF)
