# Supplementary figures and images for: Three Dimensional Culture of Human Renal Cell Carcinoma Organoids
Source: PLoS One. 2015 Aug 28;10(8):e0136758. doi: 10.1371/journal.pone.0136758 (PMC4552551; doi:10.1371/journal.pone.0136758)

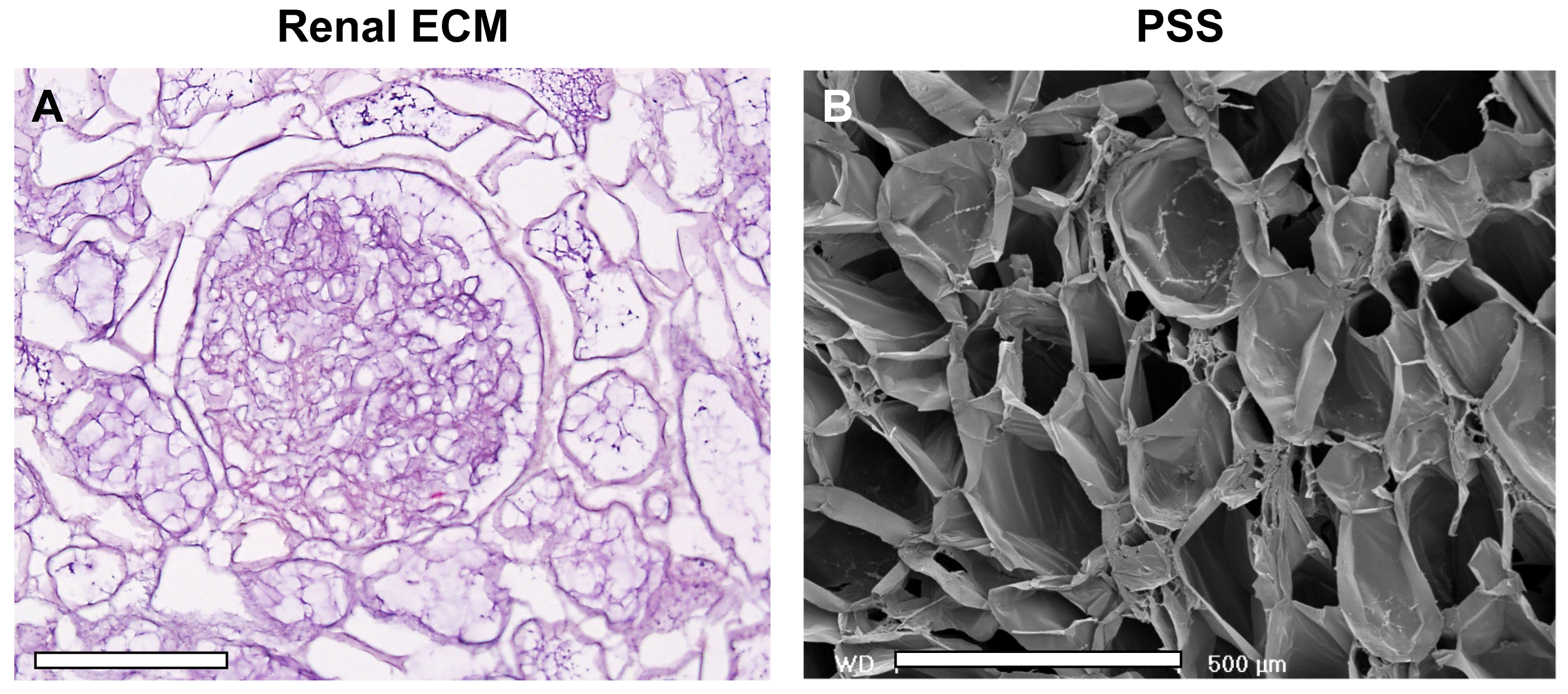

Supplement: S1 Fig — A. Renal ECM from decellularized rhesus monkey kidney retains tubular and vascular lumens as well as glomerular compartments. Scale bar = 100 μm. B. SEM of polysaccharide scaffold (PSS) with pores connected of 500–700 μm. Scale bar = 500 μm. (TIF) [file pone.0136758.s001.tif]
